# American Medical Association, Section on Stomatology

**Published:** 1901-08

**Authors:** Eugene S. Talbot

**Affiliations:** Secretary


### Reports of Society Meetings.

### AMERICAN MEDICAL ASSOCIATION, SECTION ON STOMATOLOGY.

### DISCUSSION ON SYMPOSIUM ON STATE BOARDS OF DENTAL EXAMINERS IN THEIR RELATION TO THE PROFESSION AND THE COLONIES.

*Dr. Eugene S. Talbot,* Chicago.—One important part of dental education consists in the appointment of State boards, as well as the quality of men upon these boards. A great responsibility not only rests upon them, but also great power. These men should be selected from the State dental societies. Twice the number of men required should be selected and appointed (at least the number required by law) by the governor of the State. Good men are thus secured, their appointment is legal, and it places the responsibility. The proper men can wield a great power, and they do as it is, but if they were qualified men, of high ideals, of education, the specialty would be placed where it belongs. I believe these men can control the colleges and place the entrance and final examinations exactly where they should be. That the graduates are not up to the standard has already been shown in the examinations made in the army through the National Examining Board for the army and navy. I have received a letter from Dr. Marshall in which he says forty-two graduates have been examined, and only eleven had passed. This shows that the graduates of our dental schools are certainly lacking in some particulars. This has already been noted in an editorial in one of the journals, showing great interest in the symposium of last year. Dr. Marshall's report shows that something more is lacking, and no doubt the agitation of these subjects will be productive of much good at the next meeting of the National Association in Milwaukee. This editorial hoped the men would not return to their homes without advocating the introduction of a four years' course in every dental college, and I believe the time is coming when a four years' course will be demanded, thus giving the student a firm foundation for his life career.

A four years' course will necessitate a better understanding of the subjects the teacher has under discussion. Pathology, of which the student and the profession alike know very little, will and should become one of the principal branches. The bacteriology of the mouth, a great factor in diagnosis, is really a department of dentistry, and yet the first principles are scarcely taught or understood. The etiology of diseases of the mouth is yet in its infancy, and the coming dentist must not only be a "plugger of teeth," but an all-round pathologist.

*Dr. Vida A. Latham,* Rogers Park, Ill.—I think the solving of the whole question comes with the preliminary education. Supposing the candidate enters Northwestern University, the Chicago Dental School, or any other dental college. The candidate comes in just the same as Dr. Chittenden here would come in, and is permitted to register. A man or woman comes in with a diploma from the high school in the city of A. That is a high degree for the time being; she has a good education. Another comes in from the high school in the town of C who can hardly write her own name. That has been proved over and over again. There are all kinds of high schools. You take a high school in Duluth and compare it with a high school in Chicago, or take a high school in New York and compare it with one in Holyoke,—all come in under a high school diploma; how can you make them equal or expect them to be equal? I myself had to reject candidates with a high school education. If the student does not know these common branches he cannot compete in those classes. They go before the dean and are advanced or put back, therefore they cannot do the work unless they have a good education first. That is the trouble in standardizing the elementary requirements in every case.

There is another thing to be brought out in this connection. Supposing I advertise, or that I am a quack,—I have a perfect right to be so. The State board does not take my diploma away. I can move into another State and can practise again. These are the things that should not be tolerated in this country.

*Dr. R. R. Andrews,* Cambridge, Mass.—I want to say a word in regard to raising the standard among examiners of the different States. In New England they are trying to raise the standard high enough in each State so that a student may take the examination in any State and practise without taking a further examination in any of the New England States, and that is what the profession at large is demanding. Public opinion is getting very strongly in favor of such a movement, and if the national body can adopt such a standard and live up to it, and once passing a man and passing him well and giving him the privilege of practising in any State without further examination, it will be a very desirable thing. From the fact that a man may pass a very creditable examination in all points, in bacteriology, chemistry, etc., at the time of graduating, and in five years, on account of sickness or something else, he is obliged to go to another State, he is not in condition to pass that same examination, yet he has passed five or ten years in honorable practice, and it seems to me he should be exempted and allowed to practise with that record behind him. It is to be hoped that some sort of an arrangement will be brought about sooner or later so that a man having once passed a creditable examination in any State shall be allowed to practise in any State.

There is another matter that I think will be considered in time, and that is the giving of State boards certain rights to consider cases where a man has been engaged in honorable practice for twenty or thirty years, and perhaps his wife's health or his own compels him to go West. I have in mind the case of a man who was one of the founders of the Harvard Dental School, a man who had had thirty years' active practice, and who was one of the best practitioners in Boston, who went to California. He was a man perhaps fifty-five or sixty years of age. At that time the State board had been in existence only a little while. He was called before the board and he confessed that he could not pass the examination in chemistry and bacteriology at that time, but said he could have done it when he graduated. He took the examination, however, and failed, and afterwards, in the course of two or three years, he took it again and received his license. It is hard on an old man. He is not in the condition of one who is learning and who can learn more readily, and it seems to me these cases should be considered. I have in mind a man who graduated from one of the schools in Boston. I think he failed after the three years' course and took two more years to get his degree. He is a man easily rattled by an examination, but he finally got his degree and went to Germany and practised there a number of years—I think eight or ten—with his brother. He is a fine operator. He is so fine a mechanical operator that he is to-day employed as one of the demonstrators in the schools of Boston. There is no question but that the public would be well treated if he had been given his registration. The object of registration is to protect the people from quacks. I myself am a stickler for a good, solid examination, but the examinations given by some boards, especially new men that have just come in, in some cases are pretty severe, and it has been acknowledged by some members of the board in Massachusetts that it would be very difficult for them to pass their own examinations. In fact, a member of the board of Connecticut moved to Massachusetts and tried to pass, but was turned down. I think he is studying now to pass the board, but if he does pass he will perhaps be a no better man. It seems to me, if the standard can be so raised and equalized in all the States, there will be no need to pass the examination when a man moves into another State.

*Dr. Charles Chittenden,* Madison, Wis.—I think there is scarcely a board in existence that does not consider the number of years a man has had in practice. In Wisconsin our board has in its code of rules something like this: "Candidates having had ten years or upward of practice immediately before applying for this examination shall be required to pass only a fifty per cent. examination." I think that rule applies in a majority of States.

*Dr. F. S. Dennis,* New York City.—I am not actively engaged in the work of dentistry at the present time, but the raising of the standard of dentistry is something that always appeals to me. The question came to my mind as I listened to a paper that was recently read, as to what would be the remedies for raising the standard of dental education. We have talked about dental education for years, and I believe the subject has been brought up repeatedly in this section of the American Medical Association. Is it to be remedied by law or simply by the good intent of the majority of those who have this matter in charge? By that I mean the faculties of the different schools or the members of the State examining boards.

The matter of requiring a dentist going from one State to another to pass the examination of the board of that State it seems to me is entirely wrong on the part of our examining boards. They seem to fail to recognize that years of labor and years of reputable standing in a community count for very little compared with the examination. Examinations of a dentist's ability, as a rule, do not amount to much. I think that is recognized by medical men generally. Oftentimes an examination shows but very little of what a man is capable. This is recognized by examining boards in hospitals. Often in connection with the examination they will take into consideration not merely the quality of the paper that is presented, but the quality of the man, his moral standing, his standing in the community, and his ability in lines outside of mere memory, by presenting cases to test his ability. It seems to me that a dentist who has worked for years in one State ought to be valued for his manipulative ability very much more than to have his standing judged entirely by a written paper.

In regard to the matter, advocated in one of the papers, of paying for the work of the examining board by fees from the candidates for examination, I think that method has been so thoroughly tested and proved a failure that it seems to me entirely out of the question to consider it here. The people are the ones who should bear the burden of expense. They are the ones who are being protected by the examining board, and they should bear the cost of the examination.

*Dr. J. R. Currens,* Two Rivers, Wis.—I am not a dentist, but I am a member of the examining board of Wisconsin. We had a meeting of the representatives of the examining boards of the United States yesterday in which these matters came up, and one subject impressed me here to-day, and that is in regard to reciprocity between the different boards. I think if something can be arrived at, and if our laws can be so framed that it gives boards the power to recognize licenses from other States, I cannot see but what it is the right thing to do. In the medical profession we have a little more difficulty than you dentists have. We have three or four schools to contend with, and the situation is a great deal more mixed. We have on our board two regulars, two homœopaths, and two eclectics. After the 1st of July we will have to take on one of our manipulative friends. In regard to this reciprocity business, there are many men who have grown old in the profession, whose family surroundings are such, the condition of their health is such, that they are forced to seek a climatic change, and it is a great hardship to those men to put them through an examination. The matter of diplomas and examinations came up here this afternoon. It is very hard for the State board of Wisconsin to tell about the standing of a medical college in Alabama or Tennessee. For example, these schools will publish a curriculum that will arouse admiration, but they will graduate students in four months and take almost anything in the way of entrance requirements. Under our law we can require both a diploma and examination, and we will have such under direct control. It is all right to use a diploma if it is known to be from a reputable school, but you cannot have class legislation, and there are very few boards that are allowed to use their discretion unless they have an investigation, which is a rather doubtful piece of business. Most boards have to be self-sustaining, and it would be an admirable thing if they could prevail upon the legislators to make the concession, but it is difficult to make these believe that it would be for the benefit of the people instead of the medical men and the dentists. They want us to be self-sustaining, and it places us at times in a rather bad position. There was a flaw in our law in the revision of the statutes so that we could not even send a man out to investigate a case without calling the whole board together. We had a reserve fund of twelve hundred dollars, and the result was that we had to turn it into the treasury. We tried to get it back, but could not do it. We had no money to investigate anything; the State has the money and it will stay there.

To my mind the solution of the whole matter would be this: In the first place, raise the standard high. See that the primary education is brought up to a high standard. In regard to the high school standard which has been referred to, we have a clause in our medical law which provides that the high school standard must be up to the high school standard of our State. In that way any difficulty can very easily be obviated. One medical college fought very hard against the high school standard, and we had to cut it down to the junior year, but we expect to raise it two years from now. We cannot move rapidly in these things, and in this reciprocity business we must try to get the State laws on the same plane, and if we all join hands the matter can be accomplished. The laws are queer things to handle. There is that word "reputable" that was in the law. If a man is from a reputable college the board has some discretionary powers, but if you get too particular about it the board may decide you are a little too strict. It is a hard thing to learn the ways of the law. We had a case before our board. Our law of 1897 was a very fair law. A disreputable individual took several courses in some Omaha medical college, and they refused him graduation. They pretended he did not come up to the standard. The fact was he passed a good examination, but he was refused his diploma on account of his moral character. The result was he went to St. Louis and graduated from the Barnes Medical College. He then went to North Dakota, and the secretary of the State board of North Dakota wrote to our secretary that the man was disreputable and doing all kinds of dishonorable things. He ran a lightning-rod business. He would go to a farmer with twelve children and tell him if he would give him a note for one hundred dollars he would take care of his children for twelve years. He took notes and sold them to some banks. He did the same thing in Iowa and Nebraska. Then he came to our State and wanted a license, and we refused him. We supposed our law was all right, but there was a revision of the laws in 1898. Our board feared the session laws of 1898, but supposed they were all right, but they changed the wording somewhat and the punctuation. The case came before a jury, and the fact of the matter was he beat us and we were obliged to license him. Now he threatens us with personal damage suits.

*Dr. R. H. Nones,* Philadelphia.—I am interested in the symposium and talks I have heard. There are some apparent remedies, but to select one that would answer every case is a difficult thing to do. If we do seemingly find one, it is succeeded by another problem, until we find it is impracticable. The question of how you would take care of the older practitioner if he wanted to move from one State to another, is one of the problems to be met. It would be opposed in some States that did not want a man to come over from another State, and that brings up the question of the survival of the fittest. It is a very well-known fact, yet a very sad one, that if a man has been in practice for some years he neglects many of the theoretical branches which the examining board requires him to take. Now, if that man has not taken such an examination that is demanded of the younger men, I do not see how you could get a State board to pass him. Many a young man, as has been suggested, might make a very competent dentist if he had those qualifications in later years. Why should not that young man have this same privilege to enter and bring up the necessary qualification as the older man has where you give him credit for forgetting his earlier qualifications? I say this problem and many similar problems have come to me. We have tried to get too many things instead of getting one good thing at a time. I believe, had the Association of Dental Faculties incorporated into their law a curriculum that should have been passed by the student, his preliminary education would have been much better than by grading the standard to one, two, or three years, whatever it may be, of high school. With all the careful consideration given by these gentlemen, they little thought that immediately it was entered upon the question would come up, What is one year of the high school in one State or another? Immediately that trouble arose, and it has been one of the very hardest things to contend with in colleges. I can verify Dr. Chittenden's remarks in regard to qualifications; I have seen them in all forms. When credentials have been presented to me I have invariably taken these from the students and told them I would send them to the superintendent of public instruction to have them passed upon. If a credential comes back I can follow the student, should he go to any other school, and object to his entrance upon it. The other gentleman who spoke made the point of a faculty being liable to be mandamused. That is a very strong point, indeed. You think you are treading on safe ground when your State board says to a graduate that he is not a graduate from a reputable institution. The question comes up, What is a reputable institution? In order to emphasize that point I will cite the case of the school I am connected with, the Department of Dentistry of the Medico-Chirurgical College of Philadelphia. We had some difficulty to get our dental college into the faculty. They refused us admission, and we went to teaching dentistry under the charter of the medical college. We sent our students before the State board, and they refused to examine them. We threatened them with mandamus proceedings, and after consultation with the Attorney-General they were compelled to examine our students. They passed the board, and shortly after that we were admitted to the Association of Faculties. Other States refused to do the same thing, but they soon fell into line. The men on State boards, as a rule, are very busy men, men who cannot afford to give their time to such work as this. I have seen a great deal of it. I am acquainted with the members of the State board of Pennsylvania, and I know the amount of work they have to do. I say there is absolutely no pleasure attached to it.

It is a difficult problem when the compensation of the men on the State board is to be considered. The class of men that should be on the board are difficult to obtain, because their time is of a great deal more value to themselves than the recompense they get from the State. I see no other plan except that of the fee being paid by the applicant. It is a matter of the utmost folly to ask the public to pay for such services by taxation. The general public does not desire protection. I went through one of the large department stores in Chicago, and it was a matter of surprise to me that here was being performed work in dentistry at "fair" prices. They claim to do crown- and bridge-work. If the public does not want protection in the large cities, what could you expect in the smaller towns where the people would be taxed for this protection? Would they want it? I say, most assuredly not.

There is another point that struck me while listening to these papers, and that is in regard to the matter of Dr. Marshall passing only eleven out of forty-two. That, at first thought, casts somewhat of a reflection on the various schools throughout the United States. To my mind, this would be an incorrect conclusion, for the large majority of those coming before the examining board have been several years in practice, and you can see at once that it makes it difficult for these men to pass.

I say, without hesitation, I believe the majority of the State boards of the United States could not pass their own examination. I do not think these gentlemen could have possibly kept up with the various theoretical subjects. I think it would be a point of much interest had Dr. Marshall informed us whether the failures before the army board were in the theoretical or the practical branches. I have always tried to find out the weak points of my students when they appeared before the State board, and tried to strengthen them on those points in college.

*Dr. A. H. Peck,* Chicago.—I shall not attempt to enter into a detailed discussion of the different phases of the various subjects that have been presented for our consideration this afternoon, but I do want to say that I have listened with a great deal of interest to the subject-matter presented in this symposium. Every phase is of vital importance to us, not only in dental practice, but as doctors in the dental profession, and I want to say, before proceeding further, that the work presented in the symposium last year on dental education I know has resulted in a great deal of good in dental educational circles. So I predict that the matter presented in this symposium this afternoon will in the future wield its influence for good. Personally, while I know that the officers of this section, who are especially interested in this work, are not here for the purpose of soliciting praise, yet I do feel that I want to thank them for taking up this subject and bringing it out in such a splendid manner. We must give these matters our careful personal thought and attention, we must continue to agitate them, and we must never let an opportunity pass where we can say or do anything to further the interests of higher dental education. I have always used my influence in the way of advancement of the cause of dental education in all its phases. A great deal has been presented this afternoon for our consideration, and unless an individual has given the subject a great deal of attention and thought before coming to this meeting, it is apparent that he comes here rather unprepared to discuss the subject in the most intelligent manner. I am sorry I did not have the substance of these papers to look over previously. I would have enjoyed studying them so that I might speak more to the point and possibly advance some more plausible theories or ways by which the suggestions outlined therein might be carried out. Just what is the best method of accomplishing all these desired results is quite a problem. Dr. Talbot has stated that State examining boards have power to regulate some phases of the individual college work, such as the entrance of students, preliminary requirements, etc. If Dr. Talbot's judgment is correct, and in a measure it is, I do hope the day is not far distant when the State dental examining boards may be placed in position where, by special legislation or advancement of public sentiment, they may be able to successfully use the power that is placed in their hands that the entrance of students in our various colleges may be regulated and placed in the proper condition, because we all know that the entrance of students has not been what it should be. They pay very little attention, as a rule, to credentials presented by students. Many of them are private institutions, and are in the field for private gain. This is a demoralizing condition of things, and may the day be hastened when this condition may be eliminated and our institutions placed on their proper plane.

*Dr. Charles Chittenden,* Madison, Wis. (closing discussion).— This is a subject so interesting to me that I never know where to begin nor where to stop. There is no question about the power of examining boards, provided they have the dental colleges and the dental profession at their backs. Examining boards can do anything that they desire should be done for the dental profession in the matter of standards. As I tried to exemplify in my written paper, there is no question but that the boards and the colleges together can do all there is to be done in this matter. The difficulty has been in the past with the colleges and the Association of Faculties. If the members of that Association were asked, they would admit that they have in the Association many schools that ought not to be recognized. I believe last year there was a school examined by a special committee of the Association of Faculties, and a report brought in, which I had the honor of being permitted to see, prepared to be presented to the Association, but it was so scathing in many of its criticisms that the report was withheld. I hope to see that report brought forward some day and the school properly dealt with. This Association feels that it has a lot of weak schools represented, but the disturbance of the Association is not desired. They do not seem to remember that they should come to the examiners and join hands and settle upon a standard.

The gentleman said he would like to know in what branches the students were most likely to fall down. Our experience has been that students fall down first in simple anatomy. They will fail on a dozen questions that almost any child in a common ward school could answer. That has been our general experience.

I desire to append the following letters, which speak for themselves:

"School of Dental Surgery, Wisconsin, July 29, 1899.

"Gentlemen,—Bearer, , of this city, is a young man of good moral character and a very good student. In the year 1896 he was qualified to hold a second grade certificate to teach school in , which was equivalent to a three-year course in the high school of this city.

"Yours truly,

"——— ———,

"*Superintendent of ——— School from 1894–1896.*"

"State of ———, Department of Public Instruction,

"September 24, 1900.

"Dr ———, Chicago, Ill.:

"Dear Sir,—Mr. , the bearer, impresses me as a young man in every sense qualified to take a dental course.

"He states that he completed the graded school course and a portion of the first year of the high school course in the city of .
Since then he has had several years of practical experience in a telegraph office in this city.

"Respectfully,

"——— ———,

"*Assistant State Superintendent.*"

RESOLUTIONS.

WHEREAS, Dr. John S. Marshall of Chicago, one of the most steadfast believers in the principles which this the Section on Stomatology of the American Medical Association represents, for many years past an earnest co-worker with its members in all efforts to establish the science of oral treatment upon the high plane necessary to perfect affiliation with other branches of medical science, having given evidence in the essay before us for consideration that he is carrying forward this work in practical and beneficial manner in an entirely new field; therefore be it

*Resolved,* That the secretary of this the Stomatologic Section be, and hereby is, instructed to forward to Dr. Marshall congratulations upon his appointment as Chairman of the Board of Examiners of Dentists for the Army and Navy, and also in consideration of the high standard of excellence required of applicants for examination to the end that the dental profession may be honorably and creditably represented in this branch of the service; and be it further

*Resolved,* That the members of this Section tender to Dr. Marshall at this time an assurance of their most earnest support and willingness to assist or encourage in any manner possible the furtherance of the good work he has so ably begun, the details of which have been clearly set forth in his valuable contribution to the programme of this meeting.

EUGENE S. TALBOT,
*Secretary.*

